# Smart Transportation: An Overview of Technologies and Applications

**DOI:** 10.3390/s23083880

**Published:** 2023-04-11

**Authors:** Damilola Oladimeji, Khushi Gupta, Nuri Alperen Kose, Kubra Gundogan, Linqiang Ge, Fan Liang

**Affiliations:** 1 Department of Computer Science, Sam Houston State University, Huntsville, AL 77340, USA; 2 School of Computer Science, Columbus State University, Columbus, GA 31907, USA

**Keywords:** smart transportation, internet of things, machine learning, intelligent systems, distributed systems, smart transportation applications

## Abstract

As technology continues to evolve, our society is becoming enriched with more intelligent devices that help us perform our daily activities more efficiently and effectively. One of the most significant technological advancements of our time is the Internet of Things (IoT), which interconnects various smart devices (such as smart mobiles, intelligent refrigerators, smartwatches, smart fire alarms, smart door locks, and many more) allowing them to communicate with each other and exchange data seamlessly. We now use IoT technology to carry out our daily activities, for example, transportation. In particular, the field of smart transportation has intrigued researchers due to its potential to revolutionize the way we move people and goods. IoT provides drivers in a smart city with many benefits, including traffic management, improved logistics, efficient parking systems, and enhanced safety measures. Smart transportation is the integration of all these benefits into applications for transportation systems. However, as a way of further improving the benefits provided by smart transportation, other technologies have been explored, such as machine learning, big data, and distributed ledgers. Some examples of their application are the optimization of routes, parking, street lighting, accident prevention, detection of abnormal traffic conditions, and maintenance of roads. In this paper, we aim to provide a detailed understanding of the developments in the applications mentioned earlier and examine current researches that base their applications on these sectors. We aim to conduct a self-contained review of the different technologies used in smart transportation today and their respective challenges. Our methodology encompassed identifying and screening articles on smart transportation technologies and its applications. To identify articles addressing our topic of review, we searched for articles in the four significant databases: IEEE Xplore, ACM Digital Library, Science Direct, and Springer. Consequently, we examined the communication mechanisms, architectures, and frameworks that enable these smart transportation applications and systems. We also explored the communication protocols enabling smart transportation, including Wi-Fi, Bluetooth, and cellular networks, and how they contribute to seamless data exchange. We delved into the different architectures and frameworks used in smart transportation, including cloud computing, edge computing, and fog computing. Lastly, we outlined current challenges in the smart transportation field and suggested potential future research directions. We will examine data privacy and security issues, network scalability, and interoperability between different IoT devices.

## 1. Introduction

The recent increasing urbanization is a severe multiple global problem that requires a multifaceted answer. The population living in urban areas has increased due to the increased inflow of people to the cities. The United Nations projects that the world’s urban population will reach about 4.9 billion by 2030. This raises many issues, such as pollution, traffic, resources, etc. Due to the development of Internet of Things (IoT), there are a massive number of IoT devices that are connected to the network. Those devices continuously collect data and transmit the data to computing nodes for further analysis. Due to the significant progress of deep learning techniques, many applications leverage deep learning to analyze the collected data and achieve “intelligence” and “automation”. Hence, based on the data analysis and IoT infrastructures, “Smart Cities” as a general application that includes smart grids, smart transportation, smart manufacturing, smart buildings, and much more, have become more popular [1,2,3,4].

Transportation systems are an indispensable part of people’s daily life. Since the population living in urban areas has increased, the world will thus witness explosive growth in motor vehicles, which will have a detrimental impact and contribute to traffic congestion, noise pollution, road accidents, and other issues [5]. Statistics reveal that there were around 290 million registered vehicles in the United States by the end of 2022 [6]. Furthermore, an average of 40% of the population is thought to have spent at least an hour daily on the road [7]. The increasing dependency on transportation systems has significantly increased in recent years, and thus it is common that a person in modern society has to deal with a sizable number of issues with current transportation systems on a typical day, such as traffic congestion, parking problems with limited parking spaces, longer commuting times, high levels of CO_2_ emissions, increased number of accidents, and many others.

According to estimates, traffic congestion costs the U.S. economy more than 101 billion dollars annually [8], and the economy of the European Union over 2% of GDP [9]. Moreover, as per reports published by the U.S. Federal Highway Administration, it was shown that about 50–60% of all traffic delays are the result of traffic incidents that occurred in the cities [10].

To improve the operational effectiveness of transportation systems, it is imperative to increase the use of information technology [10]. Intelligent Transportation Systems or Smart Transportation is defined as “The application of advanced sensor, computer, electronics, and communication technologies, and management strategies in an integrated manner to improve the safety and efficiency of the surface transportation system” [11]. Smart transportation systems improve traffic flow and safety, reducing travel times and fuel consumption. It is imperative to use IoT infrastructures more and seamlessly integrate information and communication technologies (ICT) to create a sustainable, intelligent transportation system. The implementation and application of cutting-edge communications, electronic, and computing capabilities enable information transfer, traffic flow control, and the administration of transportation networks. Four key concepts, sustainability, integration, safety, and responsiveness, are prioritized when adopting and implementing emerging technologies in transportation systems. These principles will be crucial in attaining the main goals of smart transportation, which are access and mobility, environmental sustainability, and economic development [12].

Smart transportation applications have a great deal of potential to address the problems faced by the constant influx of population to urban areas and deliver a safer traveling experience by extensively coordinating among various traffic control systems from different domains, operating at scale, and processing a sizable amount of data gathered from different sources. Emerging technologies will enable the sustainability of transportation infrastructures. By implementing novel techniques for gathering, processing, and disseminating information based on traffic conditions, they will encourage the efficient use of existing transportation infrastructures to regulate, control, and manage vehicular traffic. This will improve congestion management and lessen its effects [12].

This survey comprehensively examines the research on smart transportation systems and their applications, focusing on the diverse technologies utilized in intelligent transport systems. Our contributions are:

- A comprehensive survey that examines the research on smart transportation systems and their applications, with a focus on the diverse technologies utilized in smart transportation systems.
- In this survey, we assess the opportunities and challenges of deploying various technologies to smart transportation, with a particular emphasis on IoT and machine learning techniques.
- The survey evaluates communication protocols currently used in smart transportation systems while also investigating the architectures and frameworks employed in building smart transportation systems.
- Additionally, we analyze various smart transportation applications to assess the performance of communication techniques and deep learning models. We then delve into challenges associated with these technologies in smart transportation systems and recommend future research directions in the field.

### Methodology

This literature review used a formal, systematic process to identify and screen articles on smart transportation technologies and applications. For the identification of articles addressing our topic of review, we searched for articles in the four significant databases named IEEE Xplore, ACM Digital Library, Science Direct, and Springer by using the terms (“smart transportation” OR “smart transportation applications” OR “smart transportation technologies” OR “smart transportation architectures.” These terms were keyed in the databases to limit the search result to a more relevant article. Relevant articles included papers published in the past 22 years (2000–2022).

**Figure 1:**
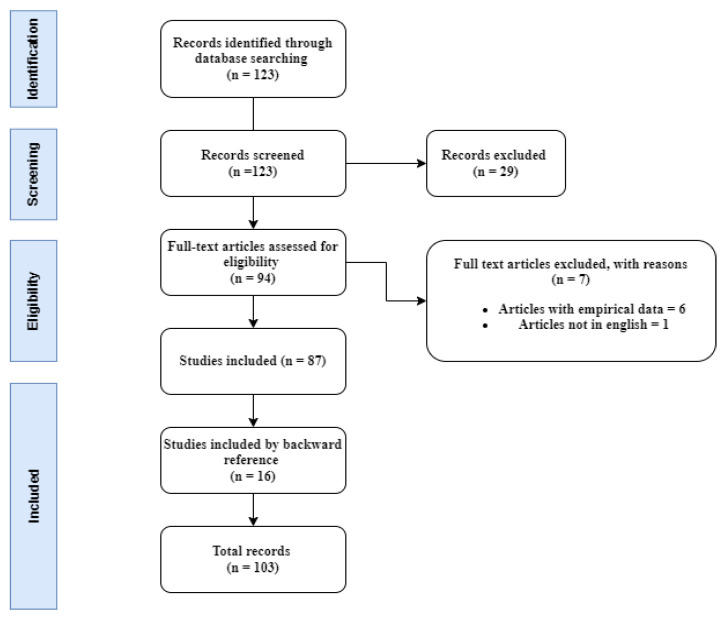
Methodology process used to identify papers reviewed (where n is the total number of papers).

A total of 123 articles from the search were screened and assessed based on the title and abstract. We excluded articles not focusing on smart transportation applications, architectures, and technologies, reducing the number of relevant articles to 94. The selected papers were downloaded and read in full. A second assessment was made based on the following criteria. The first criteria excluded articles that provide no empirical data, such as abstracts, editorials, conference summaries, short papers, and book chapters. Second, all non-English written articles were excluded. Third, similar duplicate articles were removed. After these criteria had been fulfilled, 87 articles remained. After these selections had been made and downloaded, we conducted a further backward reference search to learn more about this body of knowledge development. This method analyzed the citations in the selected articles related to the terms searched earlier. By doing that, we included 16 more articles resulting in a total of In total 103 reviewed articles, as depicted in Figure 1.

The remainder of the paper is structured as follows: Section 2 lists the different architecture and frameworks used by smart transportation systems. Section 3 presents the background of different technologies used in intelligent transportation systems, further expanding on the challenges presented by each technology. Section 4 presents the most prominent applications of ITS. Section 5 discusses the current challenges, possible opportunities, and future directions. Section 6 then concludes this paper.

## 2. Architectures and Frameworks of Smart Transportation Systems

In this section, we discuss the various architectures and frameworks adopted for developing smart transportation systems. The architectures we discuss are distributed computing, centralized computing, and edge cloud computing. Additionally, we also discuss the various communication protocols that are used in smart transportation systems.

### 2.1. Distributed Computing

Applications for smart transportation, in general, have been supported and delivered in large part by centralized computing, such as cloud computing. The cloud and networking infrastructure, however, face significant challenges in transporting and processing transportation-related data, such as CCTV streams or road sensory data, due to the constantly growing number of linked vehicles. Thus, many applications in this field call for a distributed data processing strategy instead of a centralized one due to the latency sensitivity and large volume of transportation data [13].

For instance, driving in urban settings frequently necessitates making snap judgments about whether or not to change lanes or routes to avoid traffic bottlenecks. An application must collect pertinent information, such as location, driving speeds, traffic flow, or collision events, to assist the driver in making decisions. Additionally, it must analyze those data and respond instantly. In order to meet the objectives in this scenario, the cloud infrastructure has a problem because it must quickly gather and process a large amount of data in a short amount of time. Having a distributed data processing infrastructure greatly reduces this burden of the Cloud while still achieving the latency-sensitivity requirement [14].

A wireless sensor network for Intelligent Transportation System (WITS) is a prototype for an intelligent transportation system suggested by Chen et al. [15]. The information is gathered, and the data is transferred using the WITS system. The vehicle unit, the roadside unit along both sides of the road, and the intersection unit at the intersection are the three different types of WITS nodes utilized in this system. The roadside units get the vehicle parameters from the vehicle unit, which measures them. The intersection unit then receives the information gathered by the roadside unit about the nearby vehicles. Next, the strategy sub-system determines an appropriate scheme in accordance with the predetermined optimization aim after receiving and analyzing the information from other units and passing it to the intersection unit.

CarTel is a mobile distributed sensor computing system created by Hull et al. [16]. An embedded mobile computer connected to a group of sensors is called a CarTel node. Each node collects and processes sensor data locally before sending it to a central gateway, where it is stored in a database and made available for additional analysis and visualization. CarTel generally facilitates the collection, processing, delivery, and visualization of heterogeneous data from intermittently connected mobile nodes. By gathering data on the traffic, this method may help smoothen commute times.

#### 2.1.1. Service Oriented Architectures

According to [17], (SOA) Service Oriented Architecture is a new approach to developing dependable distributed systems, one in which all the interacting components are loosely connected, and the functions are constructed as services. With all the interacting components being loosely connected and the functions being constructed as services, SOA offers an effective method for developing dependable distributed systems.

An intelligent traffic control data center for Beijing is presented by [18] and is based on SOA. The primary justification for choosing SOA for this implementation is that it includes common qualities such as distributed architecture, service-based applications, platform independence, and fine graininess. The Beijing Traffic Data Centre’s architecture aims for thorough data integration, effective data sharing, appropriate data exchange, support for on-demand services, and a cost-effective standard model for future development.

The issue of maintaining the quality of traffic information is mentioned by [19] due to the abundance of Travel Information Service (TIS) providers. This research presents the concept of a traffic information service built on SOA, which combines services or data from several sources to produce trustworthy, accurate, and comprehensive information for the traveling public. In order to collect and incorporate various types of transport information into public-oriented services, two mechanisms are therefore necessary: the first is to build a distributed architecture to integrate services from various providers, and the second is to introduce a set of uniform standards to categorize and present the description of TIS. The TIS system’s foundation in SOA enables interchange with other systems, allowing for simple integration of services from various suppliers.

The design of a GIS (Geographic Information System) transportation system built on web service technology is presented by [20]. Without the need to integrate GIS instruments, the primary goal of the GIS-T web services is to assist ITS applications with spatial data and the processing of various geoprocessing tasks, such as - detecting duplicate addresses, displaying maps, planning routes, etc. The GIS-T web service enables several transportation system departments to create a collaborative workspace, making coordination more straightforward and effective. Traditional GIS software finds accommodating all ITS requirements on a single platform difficult. However, GIS-T web services technology has provided a workable answer to this issue.

#### 2.1.2. Grid Computing

Grid computing uses numerous computer resources to work together and is loosely coupled to solve a specific problem. In grid computing, a big task is split up among numerous workstations to make the most efficient use of the resources that are available [10].

By combining the Grid, Service Oriented Architecture (SOA), and Web Service technologies, [21] introduce the Shanghai Transportation Information Service Application Grid (STISAG), a module for ITS. STISAG primarily focuses on the issue of traffic congestion in Shanghai and provides end users with a variety of real-time traffic and travel information services. The model incorporates information or services from various traffic sources, including the Shanghai Transportation Information Center, the Shanghai Taxi Company, and the Shanghai Bus Company. The model also incorporates Shanghai Grid nodes to handle and store a significant amount of traffic data as well as real-time transportation information.

#### 2.1.3. Fog Computing

Fog is defined as a network of numerous heterogeneous and decentralized devices that interact and may work together to accomplish processing and storage functions without the involvement of outside parties [22]. Fog computing can be used as a solution to the shortcomings of the cloud for smart transportation systems. The Fog computing paradigm uses the processing, storage, and network resources within the edge of the network to augment the capabilities of the cloud [23]. The Fog computing approach may be a preferable option for creating distributed applications [24] since it distributes computer resources closer to people and things, especially for latency-sensitive applications like Smart Transportation apps [25].

One of the primary application domains where the Fog Computing model exhibits the best fit, according to the authors of the original study on fog computing [23], is VANETs applications. This is especially true in light of recent developments in communication technology that enable complete Internet connectivity for infrastructure, automobiles, and other devices.

A vehicular search application using CCTVs networked in transportation infrastructure to look for a suspect driving a car is an example of a fog-based smart transportation application that can be found in [13]. An application is disseminated across various devices, including CCTV cameras, roadside units, and the like, according to a suggested programming model. To identify the vehicle in the frame, images from CCTVs are relayed to neighboring or local computing resources, like a roadside unit. If the recognition attempt fails, the computer system will send a Pan-Tilt-Zoom signal to the camera to direct its attention to a specified area to capture a clearer picture of the suspected car.

#### 2.1.4. Edge Computing

A broad definition of edge is “Enabling technologies that enable computation to occur at the network edge so that computation occurs close to data sources” [26]. The authors in the research [27] present an Edge Computing based Public Vehicle (ECPV) system to schedule ridesharing among travelers and reduce the latency of decision-making by utilizing edge computing. This system would increase traffic efficiency and vehicle occupancy ratios. In order to cut down on travel times and boost traffic efficiency, the research formalizes the public vehicle scheduling problem as an optimization problem with maximum traveler satisfaction as the goal.

### 2.2. Centralized Computing

#### Cloud Computing

Several researchers have applied various cutting-edge technologies to create smart transportation systems, but due to its sophisticated electronic data storage and communication medium, cloud computing serves as a prominent player [28]. Cloud computing gives us the ability to create and deploy computing services with minimal effort, equipment, and up-front costs [29].

Incorporating information technology, control technology, sensor technology, communication technology, and system inclusive technology, Cai and Sun [30] describe a contemporary intelligent transportation system based on cloud computing. In order to address the issues and difficulties with the current intelligent transportation system, this article presents a new generation of intelligent transportation systems based on cloud computing. From a technological standpoint, it covers the design of a cloud transportation system. From a managerial one, it shows how to develop the cloud transportation system.

Additionally, Jaworski et al. [31] proposed an urban traffic control system that uses cloud computing. Its objectives are to improve traffic flow and traffic regulation for better participant safety, less fuel consumption, and lower carbon emissions. The urban vehicle control scenario assumes that an off-board control unit that monitors each traffic intersection determines the speed of every vehicle in the controlled region. An Intersection control service (ICS) is the piece of software in charge of that. The system views the cars as cloud services, and they are found and called upon using a cloud computing technique. Targeting all vehicles in the designated zones is accomplished via geographic multicast addressing. Geographical multicast addressing uses a simple addressing mechanism by pointing to all the vehicles in a particular region. ICSs are a component of a city-wide cloud network that manages traffic flow between intersections.

On-demand bus services are demand-responsive transportation services where users can reserve their bus seats before their commute. Although on-demand bus services have been introduced in many cities, their high operating costs make them less popular. Tsubouchi et al. [32] describe their innovative solution considering the issue of high costs. Their solution is based on cloud computing technology, wherein their proposed system includes four major modules: a schedule calculation system, communication devices, a reservation interface, and a database. The primary benefit of this method is the implementation of the software blocks on remote servers. As a result, the service can be operated by the local transport authorities without them having to invest in purchasing their own servers. Thus, the system’s operational expenses are consequently decreased.

### 2.3. Edge Cloud Computing

The current Intelligent Transportation System (ITS) uses various remote sensors to assess the status of a road network in real-time. It then transmits control signals to roadside systems and road users. To transmit situation awareness and control messages, future ITS may need to communicate with users of the road network and roadside furniture. To transmit driving intention information, such as emergency braking or road conditions, vehicles may need to interact with one another. Additionally, in order to receive advanced notice of impending road conditions or to transmit control signals to controlled intersections to clear lanes for emergency vehicles and public transportation, vehicles may also need to connect with roadside equipment.

Currently, available cloud providers operate from data centers in well-connected nations. However, network latency for end users can be high due to long distances between the user and the cloud data center, and using mobile networks adds additional latency overheads. End users’ expectations are expanding to include those with wireless network connections, many of whom are actively mobile, as opposed to those who are headquartered at fixed physical locations with hard network connections [29].

By developing products based on their core cloud offerings that can run on smaller computing systems while maintaining compatibility with their core cloud platforms, established cloud vendors are beginning to experiment with edge-cloud computing.

### 2.4. Smart Transportation Communication Protocols

Modern vehicles have increasingly been equipped with a variety of sensors, actuators, and communication devices such as GPS devices, mobile devices, and embedded computers. Vehicles and nearby roadside units (RSUs) nowadays are equipped with powerful communication, sensing, networking, and processing capabilities making a vehicular ad-hoc network (VANET). They can exchange and communicate data and information with other vehicles (Vehicle—to—Vehicle network (V2V)), smart transportation devices (Vehicles—to—Infrastructure (V2I)), and applications and with the outside world using a variety of technologies (Internet of Things (IoT), Cloud computing, and distributed computing) and communication protocols (WiFi, 4G/5G, TCP/IP) which when enforced together can help researchers obtain advanced and improved transportation systems.

#### 2.4.1. 4G/5G

With the large-scale interconnections of people and things, data traffic has increased dramatically, putting pressure on the present generation of wireless mobile communication [33]. As a result of the extraordinary development in the number of connected devices, mobile data traffic, and the limits of 4G technologies, businesses, and academics are focusing their efforts on defining the standards for wireless mobile communication’s fifth generation (5G) [34].

Cellular connectivity is essential in the smart transportation system [35]. 5G is geared toward connecting individual cars through the development of Cooperative Intelligent Transport Systems (CITS). 5G can help cities become smarter by making automated transportation systems safer and more efficient than existing transportation networks. This also aids the public transportation system deal with significant transportation concerns such as traffic congestion, pollution, and crashes [33]. 5G has the potential to overcome these difficulties by creating a genuinely smart transportation system. With access to high-speed Internet on public transportation. A Linked Traffic Cloud collects and analyzes real-time data from connected cars, infrastructure, and devices to help with operational decision-making, improved navigation, fuel, and time resource optimization, and so on [36].

One of the main reasons of motor vehicle accidents is rapid traffic slowing, particularly on fast-scrolling roads and highways with poor visibility. It can be caused by other accidents, road construction, excessive motorized vehicles, particularly during peak hours, and so on. Fixed traffic sensors on roadways that connect with drivers’ mobile apps over the 4G network may often reduce such an issue, but sadly, not all roads and highways are equipped with such equipment. Celesti et al. [37] presented a possible alternative strategy for resolving such a problem by utilizing mobile traffic sensors directly mounted in private and/or public transit cars and volunteer vehicles. Experiments show that the system delivers acceptable reaction times, allowing drivers to receive alarm signals quickly, reducing the danger of potential accidents.

#### 2.4.2. V2V, V2X, V2I, V2P

**Figure 2:**
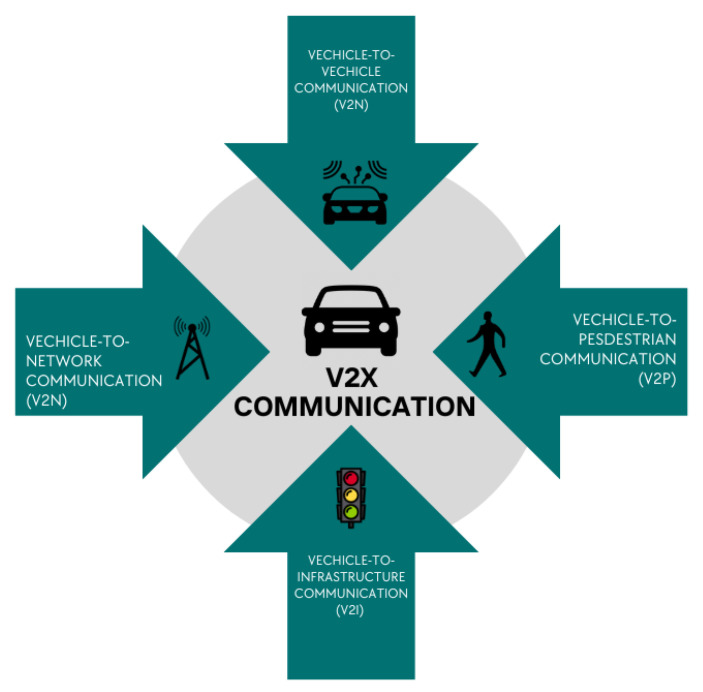
Vehicle-to-Everything Communication(V2X) protocol.

Vehicular communication use cases are classified into four categories: V2X refers to Vehicle-to-Vehicle (V2V), Vehicle-to-Infrastructure (V2I), Vehicle-to-Pedestrian (V2P), and Vehicle-to-Network (V2N) communications. V2V and V2P communications are primarily between cars or vehicles and vulnerable road users (for example, pedestrians and bicycles) to transmit position, speed, and direction information to avert accidents [38]. Figure 2 below shows the components of the vehicular communication protocol.

Direct connection between automobiles and roadside infrastructure, such as Roadside Units (RSUs), is part of V2I. The RSU acts as a forwarding node to broaden the range of communications received from a vehicle. V2N transmission occurs between a vehicle and a V2X application server, enabling services such as entertainment streaming video and connection for dynamic route management. Using a wireless network, direct or indirect communication between two cars, or between a vehicle and roadside infrastructure can increase driver safety and mobility [39]. The applications include cooperative driving assistance, decentralized probe vehicles, and user, and information communications. For example, with this technology, automobiles may broadcast a warning to other vehicles to avert accidents when changing lanes [40]. Vehicular communication, which connects cars, roadside units, and pedestrians, is a vital technology in the Intelligent Transportation System (ITS).

Concerns about location privacy and secure communication impede the adoption of smarter and safer modes of ITS. Gupta et al. [41] presented a secure and trusted V2V and V2I communication approach based on edge infrastructures, in which trusted cloudlets are used to authorize, check, and verify the authenticity, integrity, and anonymity of messages exchanged in the system rather than direct peer-to-peer communication. Moving cars or roadside equipment are dynamically linked to adjacent cloudlets, where security measures may be established to clean or prevent fraudulent communications and prevent rogue vehicles from communicating with other vehicles.

Vehicle-to-anything (V2X) communications deal with the interchanging of information between a vehicle and numerous parts of the intelligent transportation system (ITS), such as other cars, pedestrians, Internet gateways, and transportation infrastructure (such as traffic lights and signs). The technology has the potential to enable a wide range of unique applications in areas such as road safety, passenger entertainment, automobile manufacturer services, and vehicle traffic efficiency. V2X communications are now based on one of two basic technologies: dedicated short-range communications (DSRC) and cellular networks [42]. However, it is not predicted that a single technology will be able to handle such a wide range of projected V2X applications for a significant number of cars in the near future. As a result, interoperability between DSRC and cellular network technologies is recommended for efficient V2X communications.

The authors of [43] propose a reliable method for selecting relays based on distance in vehicular networks. Their approach aims to achieve fast message dissemination in a wide range of vehicle densities while also ensuring acceptable speeds in adverse scenarios. They identify the challenges of using a distance-based approach to select relay nodes in vehicular networks and present a robust relay selection method that optimizes their previous work, exponent-based partitioning broadcast protocol (EPBP). Furthermore, they provide analytical models that demonstrate the performance of their proposal in terms of contention latency and packet delivery ratio. The results revealed that the analysis and simulations across a range of vehicle densities show that their proposal has acceptable performance in adverse scenarios, improved performance in general scenarios comparable to EPBP on densely deployed networks, and superior performance in sparse densities and adverse scenarios.

#### 2.4.3. Vanet

A vehicle ad-hoc network (VANET) is defined as a group of mobile vehicles communicating over a wireless network to exchange information between themselves (V2V) and with local roadside units (V2I). This enables the dissemination of information to improve passenger safety and comfort. A VANET is a decentralized, self-organizing, dynamic network with restricted bandwidth and range only suitable for direct peer-to-peer communications [44].

Chang et al. [45] proposed three contributions to route planning utilizing VANET technology. First, a vehicular-ad-hoc-network-based A (VBA) route planning algorithm is suggested to determine the route with the shortest travel time or the lowest fuel consumption based on two real-time traffic information sources not previously employed in typical GPS applications. The initial source of traffic information is the recorded traffic information of the road segment over which the vehicle has traveled. It is then sent between vehicles through an IEEE 802.11p wireless network. Google Maps then provides the second traffic update. Finally, the VANET simulator runs simulations for six route planning algorithms in a single congested. As compared to standard route planning algorithms, VBA delivers considerable savings in both the average travel time and fuel consumption of the projected route.

Additionally, Maneguette [46] created a framework to enable various sorts of services, storage methods, access, and information management tools for diverse modes of transportation, not just for people but also for commercial vehicles and emergency services such as ambulances. Furthermore, by utilizing vehicular networks and integrating VANETs with other networks, it was feasible to enhance the capacity for abstraction to satisfy information demands, therefore providing useful information for the monitoring and administering of an intelligent transportation system.

#### 2.4.4. Wi-Fi/Wireless Sensor Network (WSN)

Smart cities increasingly use Wi-Fi connections to connect various resources. Due to its restricted bandwidth, Wi-Fi networking is typically used in smart transportation systems to connect automobiles, traffic lights, and lampposts [35]. Research organizations are proposing low-cost complementary solutions because roadside electrical equipment to support VANETs are costly. A Wireless Sensor Network (WSN) is one of the complementing solutions. The wireless self-organized sensor network nodes are often powered by battery-powered, low-cost [47], energy-efficient collection, communication, and processing technologies. These low-power nodes may often run for several years on a pair of AA batteries, decreasing maintenance requirements. Because of its low power consumption and low cost, a large number of roadside WSNs may be strategically positioned to help vehicle communication technology [48].

WSNs have lately gained prominence owing to their potential to alter many aspects of our financial system and daily lives, including shipping automation, environmental monitoring, transportation, and healthcare. The gathering and sharing of transportation information are critical in the Intelligent Transportation System (ITS). Unfortunately, most traditional ITSs can only detect a vehicle in a fixed place, and their communication and power lines raise the building and maintenance costs. The usage of wireless sensor networks (WSN) in ITSs is anticipated to address the aforementioned challenges due to its advantages, including low power consumption, wireless distribution, and flexibility without cable restrictions [49].

WSNs have assisted in many issues in transportation, such as parking automobiles which is a serious issue that contributes to traffic congestion, air pollution, and driver discomfort. Kalebe et al. [50] presented a smart parking system capable of gathering quantitative data and delivering it to drivers and other smart city apps via an expandable platform. On a three-layer architecture, the suggested system was built with wireless sensor networks for IoT-based parking. Wang and He [51] also proposed a similar solution that uses WSN for reservation-based parking policy with the potential to streamline parking system operations while also alleviating traffic congestion caused by parking hunting.

To address the difficulties of traffic control and monitoring, Sherly et al. [52] developed a real-time traffic monitoring system. The information from real-time traffic monitoring was utilized in the study to detect issues on the road. RFID, wireless sensor technologies, ad hoc networking, and internet-based information systems compose the IoT traffic architecture.

Furthermore, Sighn et al. [53] proposed a system that uses (WiFi-equipped) Smart Highways and a dashboard navigation device with a 3D camera to improve accident prevention, monitoring, and control. As a result, in the event of an accident, the video captured can be reviewed with the evidence stored simultaneously and provide additional services to capture and share real-time accident/traffic footage.

This article [54] discusses the use of a connected dominating set (CDS) as a virtual backbone for efficient routing in wireless sensor networks. Constructing a minimal CDS (MCDS) is desirable for efficient packet routing and energy conservation but is a difficult problem (NP-hard). The article proposes a new algorithm, called E-MCDS (energy efficient MCDS construction algorithm), that considers energy consumption during the construction of the MCDS. The resulting CDS is approximately composed of two independent sets (IS). The performance ratio of E-MCDS is analyzed for two different graphs and found to be 9.33opt and 17.33nkopt, respectively. Simulation results demonstrate that E-MCDS is effective in terms of both the size of the CDS constructed and energy efficiency.

Despite numerous studies that have utilized sensor mobility to enhance coverage and connectivity, little attention has been given to reducing sensor movement, which often depletes the limited energy of sensors and significantly shortens the lifespan of the network. To address this issue, Liao et al. [55] tackles the challenges of the Mobile Sensor Deployment (MSD) problem and explores ways to deploy mobile sensors with minimal movement in order to establish a Wireless Sensor Network (WSN) that offers both target coverage and network connectivity. The MSD problem is divided into two sub-problems: the Target Coverage (TCOV) problem and the Network Connectivity (NCON) problem. For TCOV, the paper presents an extended Hungarian method that can achieve an optimal solution for general cases, along with two heuristic algorithms that rely on clique partition and the Voronoi diagram, respectively. As for the NCON problem, the paper proposes an edge-constrained Steiner tree algorithm to identify the destinations of mobile sensors, followed by the utilization of the extended Hungarian method to dispatch the remaining sensors for network connectivity.

## 3. Background and Related Technologies

This section presents an overview of the technologies currently used in smart transportation and their challenges.

### 3.1. The Role of Internet-of-Things (IoT) in Smart Transportation Systems

Recent advances in wireless sensor networking, cloud computing, big data, and IoT are giving rise to a new generation of smart transportation applications. IoT consists of a network of web-enabled physical objects embedded with sensors, processors, and communication hardware that acquire data from their environments [56]. These devices form pervasive monitoring platforms that allow massive collection and exchange of real-time data, thus building the foundation of smart transportation systems.

IoT is a discovery that can solve current issues by combining technology and social implications [57]. It is a worldwide system that meets people’s demands. It allows advanced services with physical and virtual connections based on current and future developments in information and technology communication (ICT) [58]. By its name, IoT refers to the integration of data gathered from different types of objects onto any virtual platform using existing Internet infrastructure [59]. Hence, any gadget with an on/off switch that links to the internet is considered an IoT device.

IoT applications have evolved in several parts of smart transportation. Examples are smart traffic, smart parking, and intelligent mobility. These advancements make smart transportation conceivable to give drivers efficient route ideas, quick parking bookings, economical street lighting, telematics for public transportation, accident avoidance, and autonomous driving using sensors integrated into cars or mobile devices and devices deployed in the city [60].

**Figure 3:**
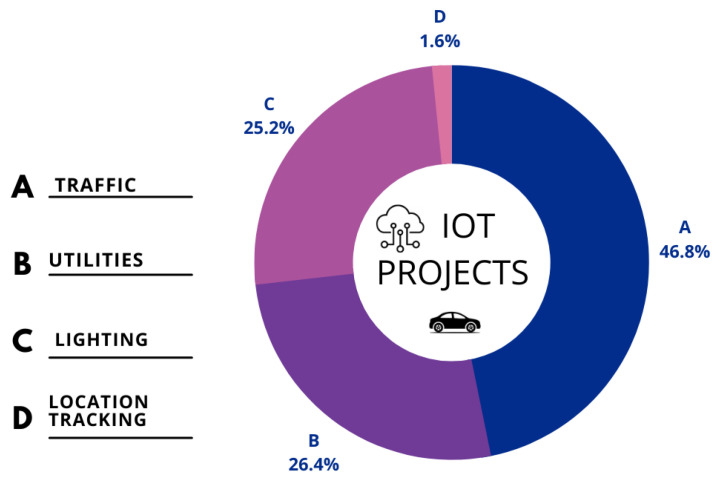
IoT transportation projects by segment in 2020.

According to IoT analytics, there is a rise in the adoption of IoT in various transportation segments. According to the 2020 research of the top IoT application categories, Manufacturing/Industrial settings are the most popular (22%), followed by Transportation/Mobility (15%) and Energy IoT projects (14%) [61]. Their research implies that the transportation industry had the second-highest use of IoT in diverse projects. Figure 3 below shows the total number of projects that leveraged IoT to provide transportation solutions.

#### 3.1.1. Existing Relevant Works

Smart transportation based on IoT technology promises to give citizens more flexible, efficient, and safe transportation options. Traffic safety is one of the significant issues that individuals face in congested cities. In this aspect, IoT can be more proactive in identifying human mistakes and preventing road accidents. Therefore, in order to achieve safer roadways, IoT-based solutions must be developed [62].

Pham et al. [63] offered a unique algorithm that improves the current cloud-based smart-parking system’s efficiency and creates a network architecture based on Internet-of-Things technology. They suggested a system that assists users in automatically finding a free parking spot at the lowest possible cost based on new performance metrics to compute the user parking cost by considering the distance and the total number of vacant spaces in each car park. The simulation results suggest that the algorithm improves the likelihood of successful parking and reduces user waiting time.

Jan et al. [64] created a model for evaluating transportation data using Hadoop and Spark to manage real-time transportation data. The data is distributed to the smart transportation system using the suggested system and decision mechanism based on the proposed data networking system. The peer-reviewed results of evaluating the suggested approach reveal data processing and real-time distribution to citizens in the shortest possible time.

Deeplaxmi et al. [65] used IoT to develop a novel system for smart transportation called Smart Vehicle Assistance and Monitoring System (SVAMS). SVAMS is an intelligent transportation system (ITS) that addresses various traffic challenges. It is a traffic management, monitoring, and optimization solution which links all cars through Zigbee and is centrally monitored and assisted by a data center. The system stores all data in the cloud for future analysis, processing, and usage. SVAMS is a low-cost, compact system with various functions such as emergency response, pollution level monitoring, automatic toll collection, traffic rule violation detection, and vehicle tracking. Adopting SVAMS will aid in developing Clean, Corruption-Free, and Crime-Free (C-3) cities.

#### 3.1.2. Challenges

IoT technology is not without peril. All connected items link together in several ways, including WiFi/Ethernet remotely through IP protocols, proximity Bluetooth for battery savings, NFC, and other medium-distance radio networks. Hidden behind it are possible dangers to data security, physical security, device security, legislation, privacy, encryption, authentication, and many other issues that must be solved so that these impediments do not obstruct future progress [66].

Several issues are now prominent due to data uploading by sensors, actuators, and intelligent appliances that post the acquired data to the internet, thereby increasing traffic. As a result, the most significant issues these systems face relates more to traffic volumes. Soltanmohamma et al. [67] explored the traffic concerns of M2M communications over LTE, concentrating on the challenges imposed on a radio access network’s access channel and traffic channel. They also outlined the benefits and drawbacks of remedies provided by other scholars. They were curious about the problems caused by M2M communication on cellular LTE and LTE-A networks [68].

Another issue is the security of IoT devices concerning smart transportation, which is critical. Most of the data acquired by IoT devices is personal and requires privacy. These sensitive data in IoT may be an open invitation for attackers to capture and consume in various ways. However, preserving privacy in IoT is challenging for several reasons. For starters, the CPU in IoT devices is restricted and cannot perform sophisticated instructions.

Additionally, the security algorithm’s power consumption must be low because most IoT devices utilize batteries. The security method’s cost should be as cheap as feasible to cover as many devices as possible. Therefore, it is better to simplify the present IP architecture to enable seamless connection and control of diverse network environments.

Other problems in developing IoT for smart transportation include device identification and addressing, mobility, interoperability, and energy efficiency [68].

### 3.2. The Role of Machine Learning (ML) in Smart Transportation Systems

A typical definition of machine learning is a system’s capacity to make intelligent choices without it being explicitly programmed. Data is at the heart of ML techniques and ML teaches computer systems to accomplish tasks like classification, grouping, prediction, pattern recognition, and many others.

The process involves training systems to archive learning by analyzing sample data with various algorithms and statistical models. It involves categorizing the sample data by quantifiable properties known as features, and an ML algorithm attempts to identify a relationship between the features and specific output values known as labels [69]. The data gathered during the training phase is then utilized to find patterns or make judgments based on new data.

**Figure 4:**
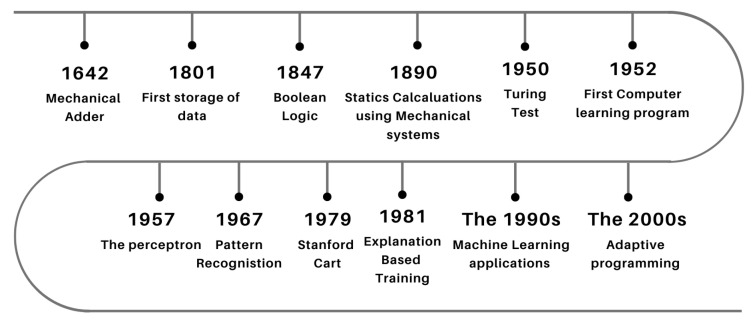
The evolution of machine learning.

Although Machine Learning has recently acquired popularity due to its exponential rate of data collection and technology improvements to enable it, its roots may be traced back to the 17th century [70]. Since the dawn of time, people have sought to make sense of data and analyze it to acquire immediate insights. Figure 4 below gives an overview of the evolution of ML.

#### 3.2.1. Existing Relevant Works

Machine learning has become the base for developing cutting-edge technology due to the speed and accuracy at which data is trained, processed, and predicted. Unsurprisingly, we now see a rise in the application of machine learning algorithms to offer solutions to some issues faced in transportation.

The widespread use of smartphones equipped with location-sensing technology has created opportunities for transportation researchers and city planners to gather detailed mobility data through smartphone-based travel surveys. By utilizing data mining and machine learning techniques to analyze this information, we can better understand the demands placed on the current commercial parking and road infrastructure. One such data collection platform that can be used for this purpose is the Future Mobility Sensing (FMS) platform. This platform can be integrated with both portable smart mobile devices and traditional GPS trackers to monitor and analyze mobility patterns. By combining raw mobility data with other relevant contextual information, machine learning algorithms can be used to infer additional trip details such as travel time, mode of transportation, and stopping points [71].

A survey was conducted in Singapore using an FMS platform to collect information on the stop activity and movement of heavy goods vehicles. However, a large portion of the recorded stops were not verified, and there was an imbalance in the reported activity types. Therefore, the authors of [71] develop a model that predicts the activity type based on various features collected through the FMS platform and point-of-interest information from Open Street Map. The proposed model uses a gradient-boosting approach and data resampling techniques to address the class imbalance. By integrating the model into the FMS platform, activity-related fields of the survey can be pre-populated to improve completion rates and reduce respondent burden. The model can also be used to recover activity information from unverified stops, providing insights into the movement and parking behaviors of commercial vehicles in Singapore.

Additionally, You et al. [72] developed a travel data collection and visualization FMS system to comprehend mobility patterns and travel behavior. The FMS system collects and fuses data from various sources and presents them visually. It is made up of two parts: (1) the FMS Data Collection Platform, which employs mobile sensing devices such as smartphones and GPS loggers, as well as machine learning algorithms with user verification to gather detailed, multi-day travel data, and (2) the FMS Data Fusion and Visualization Platform, which merges data from different sources and transforms them into knowledge for users to review their mobility diary, provide additional input and explore their mobility patterns.

To combat distracted driving, one of the leading causes of car accidents, Gosh et al. [73] created a technology similar to a high-efficiency eye blink sensor using AdaBoost, which is an Ensemble algorithm. This alarm system can eliminate several drivers distractions, perhaps leading to a road accident. In comparison, Hou et al. used four models in their paper to anticipate traffic flow for planned work zone activities. The four models are random forest, regression tree, multilayer feedforward neural network, and nonparametric regression. The results revealed that the most influential variables for highway data were the latest interval’s look-back traffic flows at the upstream, downstream, and present locations. The most relevant factors for arterial data were traffic flows from the three look-back periods at the current site only [74].

In [75], Dogru et al. compared RF to SVM and ANN to detect road accidents. SUMO [76] is used for data collecting in a traffic simulation scenario. In their research, cars communicated via a vehicle simulation to acquire information such as speed and position. The above classifiers are trained using the 10-fold cross-validation approach and verified using accuracy, sensitivity, and specificity measures. Regarding accuracy and sensitivity, the RF algorithm outperforms the other two classifiers.

This article [77] evaluated the proposed system’s performance to prior studies and several machine learning approaches, such as LightGBM, K-Nearest Neighbors (KNN), Support Vector Machine (SVM), and Random Forest. Extensive testing findings showed that the suggested system correctly recognized distinct vehicle motions with an average F1-score of 0.98, an accuracy of 0.97, and a recall of 0.98, outperforming the competitors. Furthermore, the concept is simply transferable to various drivers and locales. Since it involves the primary processing of smartphone sensor input, the system is resilient and ideal for real-time applications.

Keller et al. [78] developed a model that uses a machine learning predictive scheme in Advanced Driver Assistance Systems (ADAS) that assists drivers during emergency movements. They provided a real-time capable approach for solving the planning and control issue in a single step. Their solution works by combining a model predictive control system with an environmental model to obviate the need for a precalculated reference signal, i.e., a trajectory. The real-time capability is obtained by a crude discretization of the inputs, which allows the plant’s state trajectory to be predicted for all conceivable combinations of discretized input values.

Yu et al. [79] offered a variation-based online journey time prediction technique employing clustered Neural Networks(CNN) as input variables, using traffic vectors generated from raw detector data. They divided the corridor travel time into (1) the base term and (2) the variation term. This research conducted intense numerical tests using simulated data from the microscopic simulator CORSIM to assess the efficiency of the suggested technique.

#### 3.2.2. Challenges

The rate at which automobiles are increasing on the road is faster than the population growth rate, causing clogged highways and making it risky to travel on certain roads. It is impossible to handle this issue by expanding the number of roads since it is costly to build roads [80]. The alternative is to regulate traffic by analyzing traffic data collected on roadways; since the quantity of data created in the transportation sector is large, standard data analytics techniques may not function.

The efficacy and efficiency of a machine learning-based solution are often contingent on the data’s type and features and the learning algorithms’ performance. Collecting data in the relevant domains, such as cybersecurity and smart transportation, takes work, even though today’s internet permits the regular generation of massive amounts of data. Thus, gathering usable data for the target machine learning-based applications, such as smart city applications, and managing it is critical for future analysis. As a result, a more in-depth analysis of data collection methods is required in working with real-world data. Furthermore, historical data may contain many unclear values, missing values, outliers, and nonsensical data [81].

Machine learning algorithms significantly influence data quality and availability for training, and hence, on the final model. As a result, correctly cleaning and pre-processing the various data obtained from multiple sources is a complex undertaking. To effectively employ the learning algorithms in the related application area, it is necessary to alter or improve current pre-processing procedures or to propose new data preparation approaches [80].

Different ML algorithms exist to analyze data and extract insights. As a result, finding an appropriate learning algorithm for the intended application is complex. The complexity is because the outcomes of various learning algorithms might differ depending on the data characteristics [82]. Choosing the incorrect ML algorithm will result in unanticipated consequences, perhaps resulting in a loss of effort and the model’s efficacy and accuracy. As a result, the eventual success of a machine learning-based solution and associated applications relies heavily on the data and the learning algorithms. Suppose the data could be more suitable for learning, such as non-representative, poor-quality, irrelevant characteristics, or inadequate amount for training. In that case, the machine learning models may become ineffective or yield reduced accuracy.

### 3.3. The Role of Big Data in Smart Transportation Systems

Today intelligent vehicles and transportation systems are key technologies that enhance the convenience and security of drivers. Smart transportation systems have traditionally relied on solitary systems. Currently, these intelligent transportation systems are moving and evolving towards seamlessly integrating a wide variety of heterogeneous technologies that can gather massive amounts of data, process it, and take appropriate actions based on the data, all in real time. Due to this evolution, there is a considerable impact on the influence that a vehicle’s or driver’s behavior can have to provide numerous benefits such as preventing road accidents, reducing driver stress, reducing congestion, and many others, all of which can help regulate traffic flow throughout the city and increase information flow in case of emergencies.

Big Data has become an essential topic in academics as well as industry. It reflects vast and complicated data collection from many sources. Many prominent data processing techniques use Big Data methodologies, such as data mining, machine learning, artificial intelligence, data fusion, and social networks. Big data is a critical facilitator of smart city efforts. These two notions together may provide better services like healthcare, transportation [83], effective government, and many more, all of which improve the quality of life [84].

McKinsey Global Institute defines big data as having adequate data collecting and data management capabilities that can be utilized for large-scale and massive data storage. Big data technology has significant strategic importance, represented not only in the capacity to master vast amounts of data but also in the ability to analyze these valuable data professionally. It is an information resource that gathers, locates, and analyzes internet data to gain more helpful information.

#### 3.3.1. Existing Relevant Works

Researchers have created smart transportation applications and systems that use big data technology like the prior technologies discussed.

Wang et al. [85] and developed a systematic method for smart transportation management on bus networks in this research. A three-tiered approach is proposed to assist urban planners, managers, and technicians in their management tasks. In the system’s implementation, they used the power of Big Data. They applied Big Data techniques to compute bus travel time, and passenger demands efficiently and economically, and this system is currently deployed in Brazil.

Accidents are a significant cause of traffic congestion, resulting in fatalities and losses for those involved and lost time for those who are delayed behind the wheel. Ozbayoglu et al. [86] proposed a basic real-time autonomous accident-detection system based on computational intelligence approaches to save lives and offer faster road openings. Big data processing methods are used to populate Istanbul city traffic-flow data for 2015 from multiple sensor locations. The system predicts the chances of an accident occurring. The results show that, even while false alarms outnumber actual accident instances, the system may still give important information for status verification and early response to potential accidents.

Furthermore, Khazaei et al. [87] proposed a big Data analytic framework for urban transportation data to acquire insights into traffic patterns. The platform is cluster-based and cloud-based, providing dependability, scalability, and adaptability to changing operating conditions for online and offline analysis. It was verified using several use cases, including determining the average speed and congested stretches on major roads in the Greater Toronto Area.

Rathore et al. [88] and Babar and Arif [89] developed a graph-oriented technique to create the city’s smart transportation system. They recommended deploying road sensors to gather comprehensive traffic information and the vehicular network to obtain individual vehicle position and speed information. They presented an efficient architecture that leverages the Giraph tool with parallel processing servers to achieve real-time efficiency in processing incoming Huge Data from IoT devices, then producing big graphs from the data and analyzing them.

#### 3.3.2. Challenges

The problem in employing big data in smart transportation systems is assessing massive volumes of data and making the proper logical judgment. The difficulties of significant data-driven decision-making are widely acknowledged. Different sensors distributed across the city return massive volumes of data [84]. Precise data mining techniques are required to extract relevant information from this massive collection. When working with big data, the primary issues are data quality, data availability and connection, and processing speed [90].

- Security: One of the most challenging difficulties in any computer science application is security. Security becomes considerably more complex and difficult with Big Data. Individuals accessing data must pass through authentication mechanisms to guarantee data integrity and confidentiality. Need-based limited access is essential. Use appropriate encryption algorithms for data in transit and data at rest [91].
- Data Accuracy: Big data for smart city transportation collects real-time data from multiple sensors around the city. This data may contain noise, leading to incorrect forecasts and unneeded turmoil in intelligent city systems. Even if we analyze data fast using various hardware mechanisms, if the information used for decision-making needs to be more accurate, our efforts may be futile [92].
- Need for Speed: Companies in today’s hyper-computing world somehow need to access critical data, but they also need it quickly. Data visualization allows firms to understand and make choices more quickly, but the fundamental difficulty is the volume of data. Employing a parallel processing mechanism or a grid computing technique is possible, and it allows the organization to work in near real-time, but it is an expensive option.
- Data Interpretation: Collecting data into the correct format requires much knowledge. Data from social media is unstructured and must be preprocessed before customers and big data analytics applications can use it.
- Data Availability and Connectivity: When data for smart city and urban planning projects is collected from sensors and distributed at various sites, continuous communication with high bandwidth is required to ensure exact and timely forecast [90].
- Inadequate Skill sets: Data analyst skill is in short supply in many organizations. A team of data scientists, developers, and analysts with domain expertise is required, which is currently scarce and challenging to locate [90].

### 3.4. Autonomous Driving Systems in Smart Transportation

In smart transportation systems, autonomous driving utilizes advanced technologies, including sensors, cameras, artificial intelligence, and data analytics, which enable vehicles to operate with little or no human intervention [93]. This conveys that vehicles are only called autonomous when the automated system can carry out all alternating tasks, such as navigating through traffic in different driving environments.

To enable self-driving capabilities, these systems typically combine hardware and software components. These autonomous vehicles are equipped with hardware/sensors such as radar, lidar, and cameras to help them perceive their environment [94]. They also have advanced processing systems to analyze the data and make decisions. The software components of autonomous driving systems in smart transportation systems include algorithms and machine learning models that can interpret the data from the sensors. [95].

#### 3.4.1. Existing Relevant Works

The emergence of these self-driving technologies offers various benefits to smart transportation. Some of the benefits are improved safety, increased efficiency, greater accessibility, enhanced productivity, improved mobility, and better traffic management.

A significant benefit of an autonomous vehicle is that it can improve traffic conditions by increasing per-vehicle occupancy and decreasing the fleet of vehicles on the road [96]. Additionally, autonomous cars could perform intelligent fleet management by carefully communicating with their counterparts, reducing traffic jams. Furthermore, current autonomous vehicles have 360 degrees vision which helps reduce accidents significantly.

Anderson et al. [97] researched to prove that autonomous vehicles can reduce the poisonous gas disseminated into the air by selecting the best trip routes, consequently improving fuel efficiency. Additionally, Mobility-as-a-Service (MaaS) and car sharing are promising applications enabled by autonomous vehicles that do not require redundant human interactions. The MaaS paradigm [98] will help consumers save money, time, space, and even human resources (such as drivers). The driver of an autonomous vehicle can sit back, relax, and take pleasure in the ride. The autonomous car allows designers to create immersive passenger experiences that would not otherwise be possible [99]. Tesla is one of the current autonomous vehicles worth discussing, which utilizes a software application called “summon”. Owners of Tesla vehicles can summon their vehicles via the mobile application. Thus, the car can drive itself to a designated parking spot, such as a basement, and the owner can request to have it parked anywhere. Cars with this feature can also be parked in tight spaces where exiting is difficult [100,101].

#### 3.4.2. Challenges

Autonomous driving systems play a critical role in smart transportation systems, enhancing safety, reducing traffic congestion, and making transportation more sustainable. However, it is important to acknowledge that these systems require extensive development and ongoing innovation to address the technical, legal, and ethical issues associated with their deployment. Here, we will discuss some challenges this system faces.

- Regulation: The complexity of autonomous vehicles poses major technical challenges when it comes to their validation and testing. For traditional vehicles, current standards exist, such as that from the International Standardization Organization (ISO), which defines the functional features that their vendors must adhere to during manufacturing. However, these standards can not be applied to an autonomous vehicle because of its ability to make sole decisions, unlike traditional cars that require humans to make these decisions. Therefore, due to the high uncertainty of the predictions that these vehicles may make in different driving scenarios, it is impossible and impractical to meet all the requirements to validate and test autonomous cars [102].
- Safety and Reliability: A significant challenge for autonomous vehicles is ensuring their safety for both passengers and other road users. The implementation of autonomous vehicles can reduce accidents due to human error. However, they must be capable of detecting and reacting to unexpected situations, such as sudden changes in road conditions or pedestrian movements.
- Security and Privacy: Although promising study findings, security, and privacy remain fundamental barriers to the widespread use of connected automotive technologies. During all communications, user and location information, must be kept safe, which might be a challenge for autonomous vehicles because all of the inherent security risks connected with sensors, communication networks, and short-range communications can easily be transferred to them. As a result, security will be important in the future development of self-driving automobiles [96]. Selfish individuals, hackers, angry employees, or terrorist groups would all be interested in a completely automated system like an autonomous automobile. In the worst-case scenario, such cars may be utilized for terrorist acts without the need for a driver.
- Cost: Although tremendous progress has been made in bringing the cost of producing autonomous vehicles down, these savings are still insufficient to make them a financially viable choice for the average household. It will take some time before autonomous vehicles become a norm in middle-class families.

### 3.5. Comparative Analysis of IoT, Machine Learning, Big Data, and Autonomous Driving Services in Smart Transportation Systems

Here, we will compare the different technologies and existing literature discussed to determine which ones have been the most effective in achieving certain services. By analyzing and comparing the performance of different technologies, transportation authorities, and manufacturers can make informed decisions about which technologies and strategies to implement in their own smart transportation systems. This sub-section will explore comparative analysis in the context of smart transportation systems, highlighting some of the technologies used in the development of the smart system by various researchers and discussing some of the potential benefits/services the system targets.

In Table 1, we present a comparative analysis of the smart transportation technologies discussed previously with respect to the services they render.

## 4. Applications of Smart Transportation Systems

**Figure 5:**
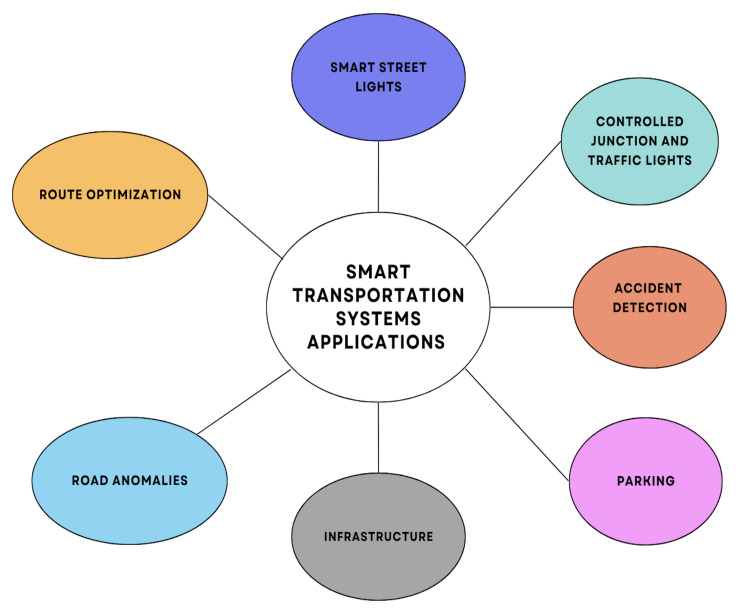
Applications of Smart Transportation Systems.

In this section, we examine the main current smart transportation systems. These systems are divided into seven classes based and their functionality as depicted in Figure 5. We divided these systems to provide a structured understanding of the different types of systems and compared their functionalities pertaining to smart transportation systems.

### 4.1. Route Optimization

Urban regions frequently have traffic congestion, which is only worsening as more vehicles are added to the road. In order to reduce traffic congestion, route optimization proposes the optimum path for a given destination. Both the amount of time it takes to travel and vehicle emissions are decreased by reducing traffic congestion [103]. The route optimization problem has been widely challenged and researched in the literature by applying various technical approaches to the IoT infrastructure.

Google was one of the first companies to harness the potential of crowdsourcing for developing new services. All modern mobile devices are compatible with the free Google Maps app. Integrated GPS, accelerometer, and gyroscope sensors are found in mobile devices. In 2009, Google unveiled a brand-new service that would provide users access to traffic data within Google Maps [104]. Fixed location sensors or other monitoring systems did not gather the traffic data. Using the maps application, the end user’s mobile device can submit anonymous information about their location and speed. To reduce congestion, Google Maps can now recommend other routes based on traffic data.

The authors of [105] investigate whether there is any connection between traffic jams, greenhouse gas emissions, and the user-generated data from Google’s “popular times” feature. In the study, cameras on particular roadways and cars with a GNSS data logger are used to collect traffic data. Emissions are estimated based on the Vehicle Specific Power (VSP) model and Google Maps’ popular time data. The findings revealed a correlation between the crowdsourced data of “popular times” and emissions, while further data calibration and an adaptive learning algorithm to examine related cases would be required to create precise correlations.

Embracing the idea of crowdsourcing, [106] used a swarm intelligence algorithm for route optimization to investigate the potential of mobile crowd-sensing for intelligent transportation systems. The authors implement a Modified Crowd-Sensing version of the Ant Colony Optimization algorithm (MoCSACO). Similar to how ants follow pheromone lines to find food, users will communicate with one another and navigate to less crowded areas by following the messages they receive from other users.

Additionally, crowdsourcing route planning for the final mile to a destination was done in [107]. According to the authors, using straightforward methods like the shortest path or shortest time rarely results in an accurate plan for the final mile. Users, on the other hand, could share their driving patterns around various destinations, providing more accurate directions at the last navigation segment. In this study, a mobile application called CrowdNavi is used to gather data and offer recommendations for a journey’s final mile. Finding the final segment is a requirement of the application. The first segment is determined using crowd data and a process known as landmark scoring, while the last portion is suggested using data from sites like Google Maps.

### 4.2. Parking

By eliminating the need to hunt parking lots in search of an open spot, making it easier to find available places in advance helps lessen traffic and pollution [29]. Many parking applications are created to monitor parking lot availability efficiently, provide users with reservation options, and even incorporate parking detection and alerting systems. Many IoT devices have been employed to detect the presence of a car in a parking spot and convey the information to a centralized system. Additionally, other studies apply ML algorithms that use image data to detect free parking slots massively. Saarika et al. [57] proposes a smart parking strategy with the concept of an IoT-supported parking lot and a smart signboard to display pertinent information. Ultrasonic sensors in the parking lot will determine whether parking spaces are available, and a WiFi module will gather and transfer the data to a cloud server. A user can now utilize a smartphone application or a smart signboard to check parking availability. The signboard is an LCD or LED display powered by a Raspberry Pi that will gather and show data on parking accessibility, weather conditions, travel times to specific locations, etc.

To determine availability, the authors in [108] also place ultrasonic sensors at each parking space. The sensor is linked to an Arduino Uno, which uses an ESP8266-01 WiFi module to transmit data to a cloud server. The MQTT protocol is used for communication. The cloud server runs ThingSpeak, an IoT platform that provides customers with various management and monitoring options. Last but not least, customers can download an Android app that enables them to reserve parking spaces and automate parking payments.

The authors suggest a smart parking system using a hardware and a software component in [109]. Magnetic sensors are employed in the hardware to detect cars in parking spaces, and a gateway device is positioned on the side of the road to collect data and transmit it to a distant server. The software component’s objective is to suggest to the user the closest available free parking space. This is accomplished using a genetic algorithm to find the shortest path between the user’s position and the closest available slot. Rizvi et al. [56] introduces an additional smart parking strategy with the development of an agent-oriented smart parking recommendation system (ASPIRE). Through a “Local Agent”, users of the ASPIRE system can configure their parking preferences, such as their favorite location and maximum walking time, and the system will take all of this information into account when a request is made for a parking space. A cloud-based software agent will thereafter select the best parking locations for the customer. The Local Agent notifies the user, who then selects their chosen parking space. The Analytic Hierarchy Process (AHP), a systematic mathematical method for analyzing complicated problems, is the foundation of the parking recommendation algorithm. RFID scanners that track and identify vehicles entering and exiting parking lots are also installed at the entrance. The cars must also be equipped with an RFID tag to make identification possible. Shi et al. [110] presents an end-to-end parking system. A smartphone application, a modular cloud server, physical sensors and microcontrollers installed at parking spaces, and a third-party payment provider make up the system. Geomagnetic vehicle detectors at the parking spaces scan for the presence of vehicles and relay the information to the cloud server using a BC95-B5 NB-IoT module. There are various modules on the cloud server. A basic information module that oversees the sensor nodes and other administrative duties a module with manageable information for the system’s maintainers, a charging module that computes charges and sends reports to the third-party charging service, and a sensor node surveillance system that allows monitoring of the availability of parking spaces and the sensor’s operational status, a business intelligence module that facilitates querying and visualizing parking data, and a task management module. Through a web-based mobile application, the system is accessible to the end user. Finally, the cloud server and the mobile web app enable payments via third-party payment services like Alipay and WeChat pay.

### 4.3. Lights

Smart Street Lights (SSL) are a crucial component of a smart city and are included in the category of smart transportation services. Smart lighting can save energy while providing dynamic functionality and manageability. Jia et al. [111] implements an SSL implementation based on IoT technology. By including a light sensor, an IR sensor, GPS, and a wireless connection module, streetlights acquire smart features. By being aware of congested locations and dynamically adjusting their light intensity, lamps can make densely populated areas safer while simultaneously using less energy. When the street light breaks, the GPS can let a centralized system tracks its location and condition and expedite maintenance procedures. The NB-IoT network serves as the foundation for the communication between the management system and SSL. The management system is built on fog nodes, which gather information from a number of bulbs and periodically assess their condition. In addition to the automatic processes that SSLs offer, they can also be remotely administered via the established management platform. Kokilavani and Malathi [112] presents a similar and simpler method for smart lights. This design connects the lamp with a light sensor, an IR sensor, and an IR led using a raspberry pi as the microcontroller. The sun’s rise and set will be detected by the light sensors, which will then turn on and off the bulb. In order to save energy, the lighting can also recognize passing vehicles or pedestrians and switch the lamps on and off dynamically.

Additionally, the authors in [113] suggest a smart lighting system in which each lamp post will function as a WiFi hotspot, allowing various types of collected data to be sent to a central web server. To achieve a considerable cost reduction, the lights will dynamically turn on/off or dim depending on the surrounding environment. Lamp posts will be embedded with cameras and sensors to monitor the area, ensuring that people are safe during crises and enhancing the capabilities of a standard lamp post.

### 4.4. Controlled Junction and Traffic Lights

A controlled junction uses traffic lights to control when vehicles may enter the junction. This is done in an effort to smooth access to a traffic jam on the route. Sensors are frequently used to control traffic signal junctions. These sensors identify areas where traffic accumulates as it approaches the junction and then extend the green light to allow for more vehicles to pass through. Transponders installed in junctions can also be used to prioritize entry to the junction so that emergency vehicles and public transportation can move through the junction more quickly. By carefully regulating the timing of traffic signals and the speed of approaching cars, intersection control tries to maximize junction throughput and reduce stopping time.

The authors in the research [114] suggest a revolutionary decentralized traffic light control system that utilizes wireless sensor networks. The wireless sensor network, the localized traffic flow model policy, and the higher-level coordination of the traffic light agents are the three levels of the system architecture. The nearest Intersection Control Agent (ICA) receives data from the wireless sensors, which track the number, speed, and other characteristics of passing cars, and uses it to estimate the intersection’s flow model. The real-time adaptive control of the traffic signals is the key contribution. This will also enhance the movement of cars. By regulating the traffic lights, an intersection control agent controls the intersection. To control a larger area, several intersection agents can communicate with one another.

### 4.5. Accident Detection

Accident detection and prevention, a domain of smart transportation, is critical for every city because an effective preventative strategy can help save lives. If drivers maintain greater concentration while on the road, accidents can be avoided. An accident prevention system allows drivers to be notified about critical situations and allow them to act promptly. By identifying accident-prone locations or accidents that have already occurred in the live traffic network, accident detection can also help to reduce the number of accidents and traffic congestion. Machine learning has shown to be particularly helpful in identifying traffic incidents, as well as in identifying patterns that may result in new accidents and alerting drivers to help them avoid them.

The research’s authors [37] suggest an IoT cloud platform to enable traffic visualization and early alerts about unexpected slowdowns that could cause accidents. The established configuration would include Infrastructure as a Service (IaaS), Platform as a Service (PaaS), Software as a Service(SaaS), and a novel approach called IoT as a Service (IoTaaS). Devices installed in volunteer vehicles will be used to collect GPS data, which is then transmitted over a 4G network to a cloud server. With the help of the OpenGTS platform and OpenStreetMaps, the cloud server manages GPS data. To facilitate further analysis, the data is stored in a SQL format and a Distributed MongoDB database. The use of Docker containers supports the scalability of the back end. The system response time is a crucial factor in the implementation’s success, and the suggested solution can transmit an alert over a distance of 1 km in a little under 120 ms.

### 4.6. Road Anomalies

Since the state of the road immediately impacts many aspects of transportation, road anomaly detection is essential in smart transportation. A road anomaly detection system’s primary function is to find potholes and bumps in the road and alert drivers. Traffic congestion, car damage, and road accidents can all result from poor road conditions.

A CNN-based method for identifying concrete fractures in photos taken with a hand-held camera in erratic lighting circumstances was proposed by [115]. The designed CNN is trained on 40 K images of 256 × 256 pixel resolutions and records with about 98% accuracy. It was reported that their suggested method was particularly effective at finding thin fractures in low-light situations that are challenging to find using more conventional techniques like Canny and Sobel edge detection.

Additionally, the use of transfer learning and pre-trained deep learning models for crack damage identification in UAV photos of civil infrastructure was proposed by [116]. (which also included a small proportion of road surface images). The results demonstrate that, without any data augmentation or preprocessing, their suggested strategy can quickly and simply achieve up to 90% accuracy in crack discovery in real-world scenarios.

### 4.7. Infrastructure

Modern transportation has profited in numerous ways from the development of IoT technology. It has developed new ways of thinking as well as new applications that have improved transportation. The capabilities of Intelligent Transportation Systems can be significantly increased by changing their infrastructure. [117] makes a novel communication method suggestion. Based on the IoT principle of M2M communication, the authors propose and simulate a vehicle-to-vehicle (V2V) communication framework. In the suggested architecture, the cars will use GPS to determine their location and communicate with nearby automobiles to exchange information about their speed, motions, and locations while simultaneously uploading the data to a server. Thus, sudden speed changes can be warned to oncoming traffic in advance to prevent accidents, and information regarding traffic congestion can be shared with oncoming vehicles to improve guidance services.

The research [118] suggests a hardware and software system to facilitate bus fleet monitoring and enhance user interaction. The suggested technology includes IR sensors to count passengers boarding and leaving the bus, RFID tags to uniquely identify buses, and GPS to track the vehicle’s whereabouts in real-time. A TI CC3200 microcontroller with an inbuilt WiFi module is used to collect and upload the acquired data to a cloud server. Additionally, each bus stop has a TI CC3200 module attached to an LCD so that the passengers can view the provided information. The users are also given access to the information via a mobile application.

The term “Social Internet of Vehicles” (SIoV) is created by fusing the concepts of social networks with the internet of things for applications in smart transportation. To reduce SIOV communication congestion, the authors of [119] suggest a cross-layered Vehicular Social Network Protocol (VSNP). To speed up communication, the protocol extends to the MAC, Physical, and network levels. Circular time slots are divided into rings by the MAC layer. Wireless Sensor Network nodes comprise the physical layer, and the network layer facilitates routing from outer rings to a fixed access point. In simulations run in Matlab, the proposed protocol outperforms an existing protocol (MERLIN).

In Table 2, we summarize some of the existing applications in smart transportation, their technologies, architectures, and communication mechanisms, along with who uses them.

## 5. Challenges and Future Direction of Smart Transportation

In this section, we highlight the present challenges with smart transportation systems as well as potential future directions for research in smart transportation systems.

### 5.1. Challenges

Although smart transportation systems have gained popularity over the years due to their ability to provide fast, convenient, and effective services, they are not without flaws.

One is the overload of wireless networks attributable to the rising number of devices used for traffic monitoring and management, which is likely the most significant recognized communication difficulty. As the number of devices increases, so will the requirement for an adaptive routing protocol for resource allocation and prioritization, as well as a system for storing and managing large volumes of road traffic data [84].

Other problems revolve around vehicle-to-vehicle (V2V) communication. New certificate management systems must be created to protect the privacy, safety, and security of infrastructure communication networks [120]. V2V communications can be the subject of transmission interference or other intrusion attempts, such as seizing control of the vehicle or manipulating data transmitted to other mobile hosts, which can lead to accidents.

With the incorporation of numerous sensors and actuators into vehicles, data collecting via a consistent process has become a new difficulty. Data collecting is also linked to data transmission to network access points (Road Side Units in most cases). To investigate, descriptions of the sensors as well as their setups are required [121].

Aside from the issue of data collection, data privacy, and security also pose significant threats to smart transportation, as discussed earlier in Section 3. Smart transportation systems create and gather massive quantities of data, including personally identifiable information. It is critical to ensure the privacy and security of sensitive data, as these systems are subject to hacking by malicious individuals for selfish reasons. These vulnerabilities could cause dire effects on passengers and drivers on the road in terms of fatalities and maybe even loss of life.

Integrating mobile (smart) devices in vehicle and transportation systems can open the way for data collection about the vehicular environment. Combining vehicle sensor data with environmental data on a computing platform is difficult since the data formats and contents differ, and there is no common technique for data fusion [90]. Interoperability can be difficult to achieve since various technologies may use distinct data formats and protocols. This can be difficult for towns and communities that do not have the appropriate technological knowledge or skill sets to maintain and equip them.

Another issue is that of costly implementation and high level of complexity within these systems. Smart transportation solutions need substantial expenditures in hardware, software, and infrastructure. This can be a significant challenge to adoption, particularly in smaller cities and villages [122]. Additionally, smart transportation systems are complicated and need specific knowledge and skills in data analytics, artificial intelligence, and IoT technology. For smart transportation systems to function efficiently, numerous technologies and platforms must be compatible.

Additionally, connectivity is paramount in a smart transportation system. To function properly, smart transportation systems rely significantly on data and communication networks. Any disruption or breakdown in these networks can result in significant issues such as traffic congestion, delays, and safety dangers.

### 5.2. Future Directions

As stated in Section 3, smart transportation is a field that focuses on improving transportation systems using advanced technologies such as machine learning, IoT, and big data analytics. Here, some fundamental open problems in smart transportation systems that require additional investigation are discussed.

- Access to the data processed and stored by these systems is crucial for government agencies, commercial businesses, and academics researching and developing new ITS technologies and services. This is because access to standardized data is critical for integrating connected and autonomous cars, as well as other sectors of this field that rely heavily on data [83,84]. Communication is a critical component of data access when cars and travelers traverse jurisdictional boundaries and they are critical for the next generation of smart transportation systems. Research could be conducted into ways to increase the resilience of smart transportation systems through backup systems, redundancy, and disaster recovery planning.
- Another significant challenge is security and privacy; these are critical and continuous issues in the transportation industry [91]. Cyber attacks on transportation infrastructure can impact national security, public safety, and the economy. Future research can explore ways to improve the security of data transmission, storage, and processing, including encrypting, controlling access, and detecting intrusions. Additionally, the use of advanced authentication and authorization systems, intrusion prevention systems, and threat intelligence can be explored in future research on smart transportation systems.
- Exploring the potential of autonomous cars to decrease traffic congestion and increase road safety: As stated earlier, autonomous cars can alleviate traffic congestion while also improving road safety [96]. Researchers may look into how effective these cars are in reducing traffic congestion and improving road safety, as well as their effects on transportation demand and the environment.
- Investigating the use of blockchain technology to improve transportation security, efficiency, and reliability: By providing a secure and transparent record of transactions, blockchain technology can improve transportation security, efficiency, and dependability [123]. Researchers may examine blockchain technology’s potential in smart transportation and create new applications and use cases.
- Examining the role of smart transportation in enhancing accessibility and mobility for disadvantaged groups (e.g., the aged, the disabled, and the low-income): Smart transportation may increase accessibility and mobility for underserved populations, including the elderly, disabled, and low-income people, [96]. Researchers may explore the efficacy of smart transportation solutions in enhancing accessibility and mobility for these populations, as well as create methods to overcome any adoption hurdles.
- Green transportation: Smart transportation systems can be structured to prioritize and encourage environmentally friendly modes of transportation [97] such as public transportation, cycling, and electric automobiles. This would assist in cutting greenhouse gas emissions while also improving air quality in cities.

## 6. Conclusions

In this paper, we gave an in-depth survey of smart transportation systems and applications by reviewing the major technologies currently used to develop smart transportation systems. We highlighted the challenges, history as well as smart transportation applications using these technologies. Additionally, we discussed the common architectures that provide the development schemes of smart transportation systems. We assembled several current research relating to these technologies as well as theoretical evaluations of these systems. We then aggravated the uses of these applications into the segments that are applied in the smart transportation sector.

Furthermore, the communication protocols used by these smart transportation systems to ensure functionality were analyzed. Common communication protocols were explained, as well as systems that currently use these protocols to improve the issues of the transportation systems. The challenges currently facing these systems have been addressed in this paper as well as future research that could have benevolent impacts in improving the issues of these systems in our society. In this paper, we gave an in-depth survey of smart transportation systems and applications by reviewing the major technologies currently used to develop smart transportation systems. We highlighted the challenges, history as well as smart transportation applications using these technologies. Additionally, we discussed the common architectures that provide the development schemes of smart transportation systems. We assembled several current research relating to these technologies as well as theoretical evaluations of these systems. We then aggravated the uses of these applications into the segments that are applied in the smart transportation sector.

Furthermore, the communication protocols these smart transportation systems used to ensure functionality were analyzed. Common communication protocols were explained, as well as systems that currently use these protocols to improve the issues of the transportation systems. The challenges currently facing these systems have been addressed in this paper and future research that could have benevolent impacts in improving the issues of these systems in our society.

## Figures and Tables

**Table 1:** Comparative Analysis of Smart Transportation Technologies and their Services.

Services	Benefits	Papers	Technology Used
Mobility as a Service(MaaS)	Real-time data analytics for car informatics Transportation services via cloud computing	[63,64,77,85,87,88,89,98]	IoT, Machine learning, Big data, Autonomous systems.
Shared Autonomous Vehicles (SAVs)	It combines the benefits of shared mobility and autonomous vehicles to provide a safe, convenient, and cost-effective transportation solution.	[96,97,98,99]	Autonomous driving systems, Machine learning
Incident management systems (IMS)	Manages events that occur on transportation systems, such as accidents, breakdowns, or other disruption. Proactively manages traffic flow	[65,72,75,76,78,86]	IoT, Machine learning, Big data
Shared mobility	Ride-sharing services Reduced traffic congestion Improved air quality Increased accessibility to transportation options	[85,88,97]	Big data, Autonomous driving system
Location Tracking Systems	Employs GPS technology to track the position of vehicles Enables manufacturers, or car owners to track the performance of their vehicles in real-time	[71,79,88]	Machine learning

**Table 2:** Summary of Existing Applications.

Application	Technology	Architecture	Communication Mechanism	Used by: End User/Management
[41]	IoT (V2V)	-	-	End User
[42]	IoT	Cloud	4G	End User
[58]	IoT, RFID	Cloud	3G/4G	End user
[109]	Crowd Sourcing	Cloud	4G/5G	End User
[110]	Crowd Sourcing	-	-	End User
[111]	Crowd Sourcing	Client - Server	-	End User
[112]	Crowd Sourcing	Cloud	3G/4G,	End User
[113]	IoT	Cloud	Wireless Sensor Network	End User
[114]	IoT	Cloud	Wireless sensor network	End User
[115]	IoT	Cloud	4G	Management
[116]	IoT	Fog Computing	Wifi, 4G	Management
[117]	IoT	-	-	Management
[118]	IoT (RFID, GPS)	Cloud	Wifi	End User
[119]	IoT	Wireless sensor neworks	Vehicular Social Network Protocol	Management
[120]	Machine learning (CNN)	-	-	Both
[121]	Deep Neural Network	-	-	Both

## Data Availability

The data that support the findings of this study are openly available in Google Scholar.
